# Supplementary material for: Entropy of Simulated Liquids Using Multiscale Cell Correlation
Source: Entropy (Basel). 2019 Jul 31;21(8):750. doi: 10.3390/e21080750 (PMC7515279; doi:10.3390/e21080750)
Supplement: Supplementary file 1 [file entropy-21-00750-s001.pdf]

Supplementary Information for
Entropy of Simulated Liquids Using Multiscale Cell Correlation

by Hafiz Saqib Ali, Jonathan Higham and Richard H Henchman

Table S1. Symmetry Number for Each Molecule

Liquid	σ	Axes number	Liquid	σ	Axes number
acetic acid	1	3	formic acid	2	3
acetone	2	3	furan	2	3
acetonitrile	1	2	hexane	2	3
ammonia	3	3	hexanol	1	3
aniline	2	3	hydrazine	2	2
benzene	12	3	hydrogen peroxide	2	3
benzyl alcohol	2	3	hydrogen sulfide	-	0
benzaldehyde	2	3	methane	-	0
butane	2	3	methanethiol	1	2
butanol	1	3	methanol	1	2
2-butaoxyethanol	1	3	methylamine	1	2
carbon dioxide	2	2	NMA	1	3
chloroform	3	3	octanol	1	3
cyclohexane	6	3	pentane	2	3
diazene	2	2	pentanol	2	3
dichloromethane	2	3	piperidine	1	3
diethanolamine	2	3	propane	2	3
diethyl ether	2	3	propanol	1	3
DMFA	1	3	pyridine	2	3
DMSO	2	3	styrene	1	3
1,4-dioxane	2	3	TBA	3	3
ethane	2	2	TFE	4	3
ethanol	1	3	tetrahydrofuran	1	3
ethene	2	2	toluene	2	3
ethyl acetate	1	3	triethylamine	3	3
ethylamine	1	3	m-xylene	2	3
ethylene glycol	2	3	o-xylene	2	3
formamide	1	3	p-xylene	4	3

Table S2. Experimental Data to Calculate Liquid-Phase Entropy

Molecule	$S_{\text{gas, bp}}$ / J K^{-1} mol^{-1}	ΔH_{vap} / kJ mol^{-1}	$C_{\text{P,gas}}$ / J K^{-1} mol^{-1}	P_{vap} / kPa	Equation
ethylamine	284.8[b]	28.0[a]	72.6[b]		$S^{\text{o}}_{\text{liq}} = S^{\text{o}}_{\text{gas}} + C_{\text{P}} \ln \frac{T_{\text{vap}}}{298} - \frac{\Delta H_{\text{vap}}}{T_{\text{vap}}}$
triethylamine	405.4[b]	35.1[a]		7.66[a]	$S^{\text{o}}_{\text{liq}} = S^{\text{o}}_{\text{gas}} + R \ln \frac{100}{P_{\text{vap}}} - \frac{\Delta H_{\text{vap}}}{298}$

[a] Ref 1.
[b] Ref 2.

Table S3. MCC Entropy Components (J K^{-1} mol^{-1}) for OPLS and GAFF

Liquid	$S_{\mathrm{M}}^{\mathrm{transvib}}$		$S_{\mathrm{M}}^{\mathrm{rovib}}$		$S_{\mathrm{M}}^{\mathrm{topo}}$		$S_{\mathrm{UA}}^{\mathrm{transvib}}$		$S_{\mathrm{UA}}^{\mathrm{rovib}}$		$S_{\mathrm{UA}}^{\mathrm{topo}}$	
	OPLS	GAFF	OPLS	GAFF	OPLS	GAFF	OPLS	GAFF	OPLS	GAFF	OPLS	GAFF
acetic acid	58.3	58.5	52.0	51.8	30.0	29.5	6.7	12.2	29.6	28.2		
acetone	65.4	67.3	58.3	59.5	25.2	23.5	7.4	11.3	45.9	44.7		
acetonitrile		63.9		38.7		16.0		1.2		22.7		
ammonia	34.8	41.7	14.5	20.4	22.0	21.1						
aniline	66.7	67.0	56.1	56.2	26.6	26.9	16.1	16.8	39.5	38.3		
benzene	73.1	74.1	61.5	61.4	11.7	10.8	7.9	8.6	28.7	27.0		
benzyl alcohol	68.2	66.4	59.4	57.6	22.1	21.9	22.4	22.7	43.6	39.8		
benzaldehyde	70.2	71.6	60.0	59.9	22.9	21.5	21.5	23.6	29.2	27.4		
butane	67.7	68.5	57.1	58.7	23.7	23.6	10.8	8.7	51.3	45.1	3.7	6.9
butanol	64.1	62.9	54.8	53.9	28.9	29.2	22.6	20.2	55.7	51.2	17.9	17.9
2-butaoxyethanol	64.9	65.8	55.3	59.4	24.1	23.6	60.7	54.8	72.6	67.3	15.6	29.7
carbon dioxide	53.6	50.3	42.3	40.4	13.6	14.1	1.5	1.5				
chloroform	80.6	81.5	77.3	77.4	21.3	21.3	20.7	25.3	3.3	4.4		
cyclohexane	70.2	70.1	64.2	64.2	19.0	19.2	13.3	11.6	53.4	46.8		
diazene	57.6	54.9	32.4	30.8	11.6	12.2	0.0	0.0	23.4	18.2		
dichloromethane	80.3	79.5	71.6	71.4	23.4	23.4	6.8	6.4	8.2	10.2		
diethanolamine	58.4	58.7	48.1	53.2	21.5	18.1	46.7	42.9	65.6	63.7	7.9	19.9
diethyl ether	69.3	69.7	58.8	59.4	21.9	21.5	23.1	21.9	56.5	55.9	7.7	8.0
DMFA	64.6	64.8	56.7	57.2	30.6	29.9	13.3	22.3	48.5	47.9		
DMSO	62.1	66.2	54.8	58.5	25.6	25.9	7.9	12.1	32.5	39.6		
1,4-dioxane	66.6	66.5	61.3	61.0	27.6	27.9	13.7	13.0	36.4	30.6		
ethane	55.0	56.6	30.8	32.0	12.2	12.0	0.0	0.0	27.1	26.6		
ethanol	60.7	59.7	48.7	48.2	27.8	28.0	2.9	2.6	36.9	37.0		
ethene	54.5	58.8	29.7	31.8	12.2	11.2	0.0	0.0	17.3	17.8		
ethyl acetate	66.1	67.7	58.7	59.9	28.8	27.5	35.4	33.3	51.0	49.3	14.4	13.9
ethylamine	59.8	62.0	47.4	50.9	29.8	29.6	2.2	2.3	41.0	40.0		
ethylene glycol	57.0	56.5	45.2	48.5	26.1	24.6	8.7	10.9	35.2	34.1		
formamide	56.7	56.3	44.1	45.8	31.6	31.8	1.1	1.0	17.2	18.4		
formic acid	61.1	56.6	48.3	43.5	30.5	32.1	1.3	1.0	14.8	11.7		
furan	69.5	71.7	60.1	62.2	27.0	25.3	7.5	7.1	18.5	19.3		
hexane	70.7	71.2	60.7	62.2	20.1	20.8	34.9	30.9	75.1	68.7	11.5	18.0
hexanol	65.7	64.7	56.7	56.2	25.5	26.0	45.8	41.9	73.3	66.5	20.5	26.1
hydrazine	51.4	50.0	26.4	26.9	12.0	12.1	0.0	0.0	30.4	26.9		
hydrogen peroxide	54.0	53.8	29.0	30.4	24.1	22.7	0.0	0.0	18.7	18.0		
hydrogen sulfide		60.6		40.4								
methane	40.2	42.8	33.0	35.3								
methanethiol	71.0	71.4	43.7	43.6	16.9	16.1	0.0	0.0	45.3	41.3		
methanol	58.9	58.9	32.7	32.7	14.6	14.6	0.0	0.0	33.0	33.0		
methylamine	53.0	55.8	27.6	31.0	15.8	16.4	0.0	0.0	31.3	30.3		
NMA	59.7	61.7	52.3	54.8	28.4	27.6	13.7	13.3	50.5	48.8		
octanol	66.4	65.7	57.3	57.5	22.7	23.4	71.9	66.0	90.6	81.9	26.0	36.3
pentane	70.8	71.4	60.8	62.2	21.7	22.3	22.9	20.4	66.4	61.2	7.9	12.6
pentanol	65.1	63.8	56.0	55.2	27.2	27.6	33.8	30.7	64.5	58.9	16.9	21.0
piperidine	67.8	67.0	61.0	59.6	32.9	32.6	11.8	11.5	60.8	51.5		
propane	62.2	63.3	51.5	52.8	25.6	25.5	1.2	1.0	35.7	33.7		
propanol	62.9	61.6	52.9	51.8	29.5	29.5	12.1	11.1	47.0	43.7	8.9	8.9
pyridine	69.9	70.5	59.4	60.0	27.2	25.5	8.8	9.6	25.2	23.5		
styrene	73.4	75.7	61.6	61.7	29.6	28.7	19.8	21.7	38.6	35.2		
TBA	64.1	63.1	57.2	56.3	20.1	19.3	16.8	15.1	60.1	63.7		
TFE		77.0		70.0		18.5		41.6				
tetrahydrofuran	69.5	71.8	60.1	62.3	32.7	31.1	7.5	7.1	18.5	19.3		
toluene	72.7	74.0	61.4	61.8	25.2	23.9	16.9	17.7	47.7	45.8		
triethylamine	69.4	69.9	63.2	63.7	21.9	20.9	38.7	38.6	79.3	75.5	19.2	26.1

m-xylene	71.8	73.5	61.2	62.1	24.3	22.6	24.6	25.5	66.3	64.3
o-xylene	73.6	75.1	61.6	62.2	24.9	23.4	23.2	23.9	62.0	58.4
p-xylene	71.8	73.7	60.8	61.7	18.4	16.5	25.6	26.3	66.2	64.3

Table S4. Conformation Probabilities[a]

Liquid	p_t		p_{g-}		p_{g+}	
	OPLS	GAFF	OPLS	GAFF	OPLS	GAFF
butane	0.88	0.69	0.88	0.15	0.06	0.15
butanol	0.24	0.23	0.43	0.43	0.43	0.34
(C terminus)	0.27	0.28	0.35	0.35	0.38	0.37
2-butoxyethanol	0.79	0.71	0.10	0.15	0.10	0.15
(C terminus)	0.94	0.51	0.04	0.24	0.03	0.25
	0.86	0.88	0.07	0.06	0.07	0.06
	0.84	0.86	0.08	0.07	0.08	0.07
	1.00	0.04	0.00	0.48	0.00	0.48
diethanolamine	1.00	0.10	0.00	0.50	0.00	0.41
	0.88	0.94	0.06	0.03	0.06	0.03
	0.84	0.95	0.09	0.03	0.07	0.03
	1.00	0.09	0.00	0.43	0.00	0.47
diethyl ether	0.87	0.86	0.07	0.07	0.06	0.07
	0.87	0.86	0.06	0.07	0.07	0.07
ethyl acetate	1.00	1.00	0.00	0.00	0.00	0.00
(acetate C-terminus)	0.00	0.00	0.50	0.50	0.50	0.50
	0.51	0.58	0.25	0.21	0.25	0.22
hexane	0.87	0.73	0.07	0.13	0.06	0.13
	0.87	0.77	0.07	0.12	0.06	0.11
	0.86	0.75	0.07	0.13	0.07	0.12
hexanol	0.86	0.75	0.07	0.13	0.07	0.12
(C terminus)	0.87	0.76	0.07	0.12	0.07	0.11
	0.87	0.77	0.07	0.12	0.07	0.11
	0.43	0.51	0.29	0.24	0.28	0.25
octanol	0.88	0.75	0.06	0.13	0.06	0.13
(C terminus)	0.89	0.79	0.05	0.10	0.05	0.12
	0.90	0.80	0.05	0.10	0.05	0.10
	0.89	0.78	0.06	0.11	0.06	0.12
	0.88	0.77	0.06	0.11	0.06	0.12
	0.44	0.52	0.30	0.24	0.26	0.24
pentane	0.86	0.74	0.07	0.13	0.07	0.13
	0.86	0.73	0.07	0.14	0.07	0.14
pentanol	0.85	0.70	0.08	0.15	0.08	0.15
(C terminus)	0.87	0.77	0.07	0.11	0.06	0.11
	0.44	0.50	0.28	0.25	0.28	0.25
propanol	0.24	0.23	0.43	0.44	0.33	0.33
triethylamine	0.43	0.39	0.11	0.27	0.46	0.34
	0.43	0.39	0.09	0.25	0.49	0.36
	0.43	0.39	0.08	0.27	0.48	0.34

[a] Dihedrals are ordered sequentially from the terminus given.

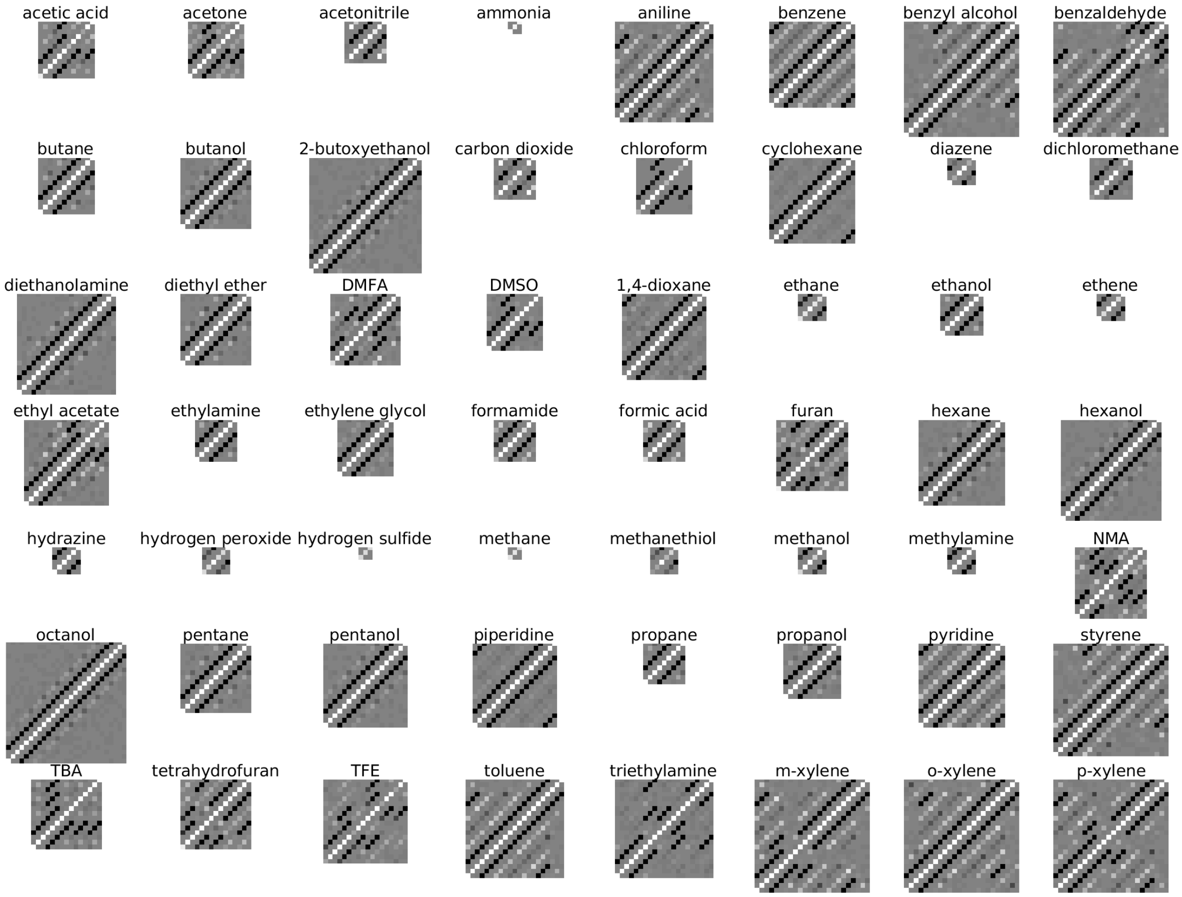

Figure S1. United-atom (UA) force covariance matrices for each liquid (GAFF), with the origin at the lower left. White and black represent correlations of 1 and −1, respectively, with grey in between.

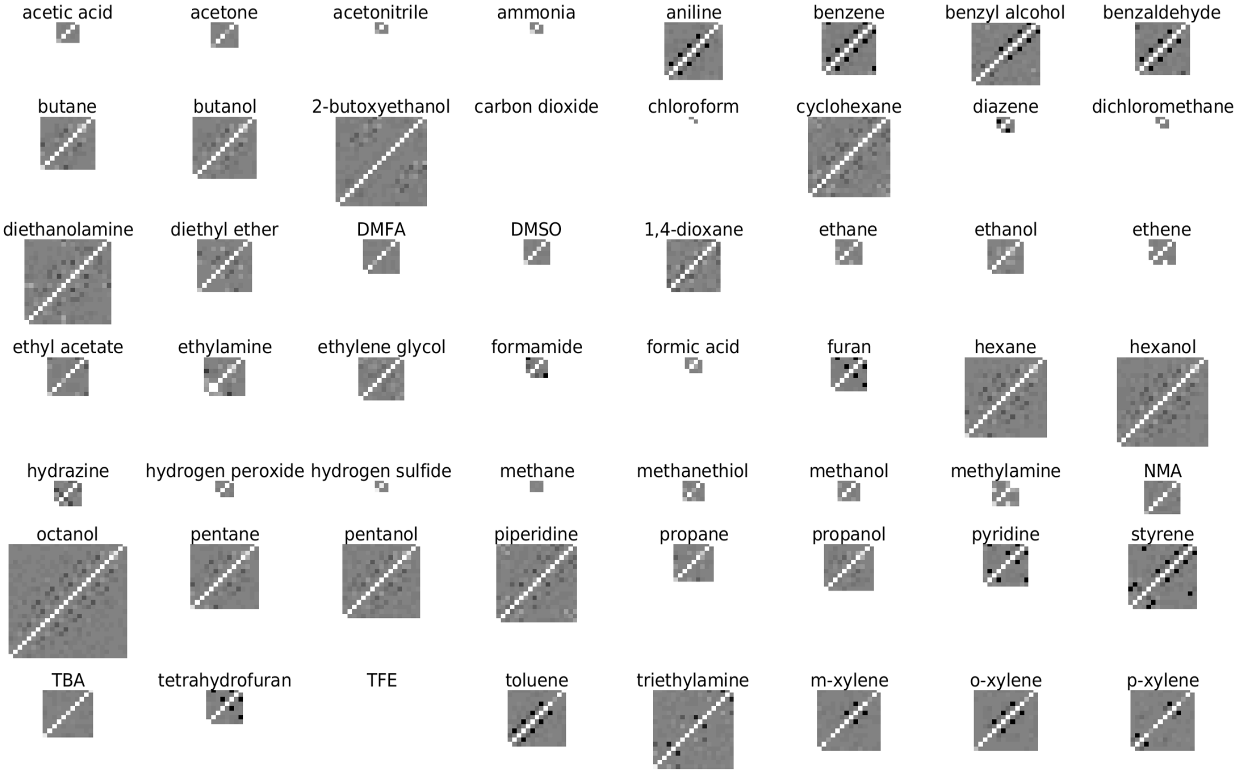

Figure S2. UA torque covariance matrices for each liquid and otherwise as for Figure S1.

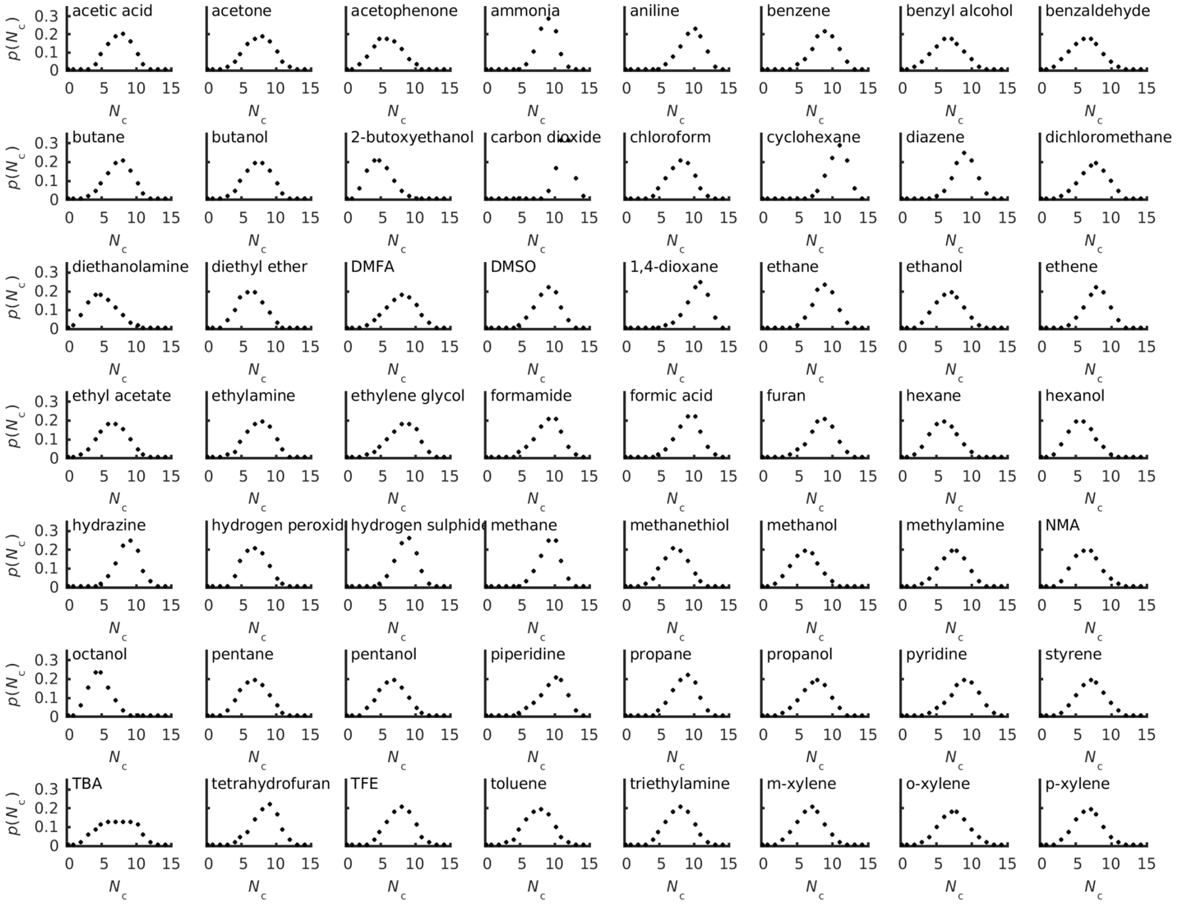

Figure S3. Probability distribution functions $p(N_c)$ of coordination number N_c for each liquid (GAFF).

References.

1. NIST Chemistry Webbook, Standard Reference Database Number 69, National Institute of Standards and Technology, http://webbook.nist.gov/chemistry/.

2. Stull, D.R.; Westrum Jr, E.F.; Sinke, G, C. *The Chemical Thermodynamics of Organic Compounds*; Wiley, 1969.
